# Supplementary material for: Identification of photoperiod-induced specific miRNAs in the adrenal glands of Sunite sheep (Ovis aries)
Source: Front Vet Sci. 2022 Jul 22;9:888207. doi: 10.3389/fvets.2022.888207 (PMC9354845; doi:10.3389/fvets.2022.888207)
Supplement: Supplementary file 1 [file Data_Sheet_1.ZIP › Supplementary file/miRDeep2 file.zip!/miRDeep2 file/Novel_Structure/LP42A3/Novel_661.pdf]

Provisional ID	:	NC_019458.2_350
Score total	:	2.7
Score for star read(s)	:	3.9
Score for read counts	:	-4.4
Score for mfe	:	1.6
Score for randfold	:	1.6
Score for cons. seed	:	
Total read count	:	3
Mature read count	:	2
Loop read count	:	0
Star read count	:	1

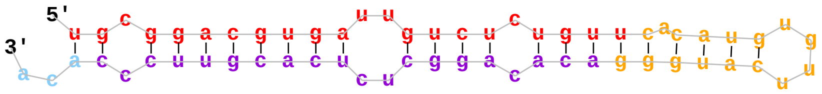

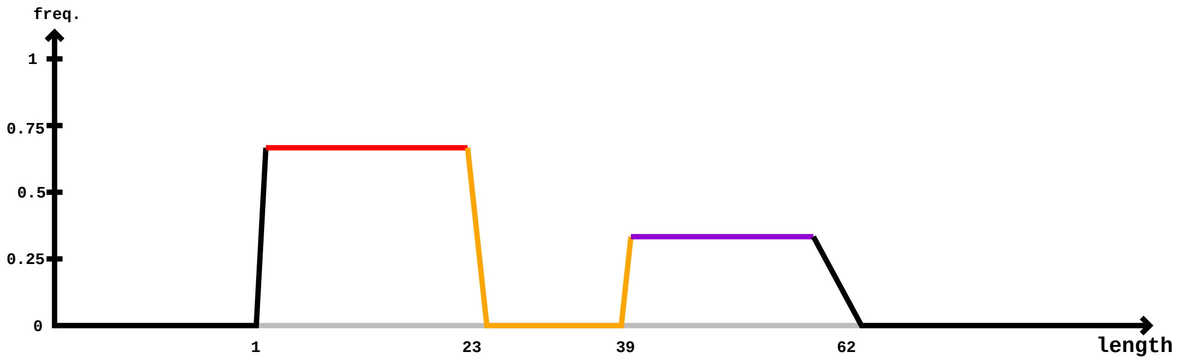


```
                      Mature                                        Star
5'- agcuucugguccucaggcaaugcggacgugauugucucuguucacauguguucaugggacacaggcucucacguucccacacuugaccuugaucuuguccuuguuuacaagg   -3'   obs
    agcuucugguccucaggcaaugcggacgugauugucucuguucacauguguucaugggacacaggcucucacguucccacacuugaccuugaucuuguccuuguuuacaagg         exp
    .......((((...((((((((.((((((((..((((.(((((.((((....))))))))).))))..)))))))).))...))).))).))))....(((((....)))))   reads   mm   sample
    ....................ugcggacgugauugucucuguu......................................................................   1       0    seq
    ....................ugcggacgugauugucucuguuc.....................................................................   1       0    seq
    ..........................................................acacaggcucucacguuccc..................................   1       0    seq
```
